# Sleep disturbances in brain tumors: a narrative review

**DOI:** 10.3389/fonc.2025.1594232

**Published:** 2025-09-25

**Authors:** Qiang Liang, Tianyi Hu, Tong Yang, Yawen Pan, Karen Spruyt, Xinyan Zhang, Qiang Li

**Affiliations:** ^1^ Department of Neurosurgery, Lanzhou University Second Hospital, Lanzhou, China; ^2^ Academician Workstation of The Second Hospital & Clinical Medical School, Lanzhou University, Lanzhou, China; ^3^ The Second Medical College of Lanzhou University, Lanzhou University, Lanzhou, China; ^4^ The First School of Clinical Medicine, Lanzhou University, Lanzhou, China; ^5^ Université Paris Cité, NeuroDiderot, INSERM, Paris, France; ^6^ Department of Neurology, West China Hospital, Sichuan University, Chengdu, China

**Keywords:** brain tumor, sleep disturbances, insomnia, narcolepsy, prognosis

## Abstract

Individuals with brain tumors are more susceptible to comorbid sleep disturbances, which significantly impair daytime functioning, quality of life, and long-term prognosis. A bidirectional relationship between sleep and brain tumors has been suggested, with sleep disturbances in this population being diverse and multifactorial, stemming from neurotransmitter imbalances, treatment interventions, and comorbidities conditions throughout the disease course. While sleep assessment and intervention guidelines exist for cancer more broadly, specific recommendations for neuro-oncological etiology populations remain limited. As awareness grows regarding the negative impact of poor sleep in patients with brain tumors, there is an urgent need for more targeted research to systematically characterize sleep disturbances and explore therapeutic implications. In this context, we conducted a narrative review current on sleep research in patients with brain tumors.

## 1 Introduction

Brain tumors include primary neoplasms arising from cerebral cells or metastatic lesions in the central nervous system (CNS). The average age-adjusted annual incidence rate of CNS tumors is 24.7 per 100,000 population (1), accounting for approximately 1.4% of all cancers. Among these, benign brain tumors, such as meningiomas and craniopharyngiomas have an incidence of 17.69 per 100,000, while malignant tumors, including gliomas and metastases, occur at a rate of 7.02 per 100,000 (1). Despite advances in treatment, the five-year overall survival rate following surgery for brain tumor patients remains below 30% (2).

The development of brain tumors is influenced by various factors, including genetic predisposition, radiation exposure, and viral infections. Symptoms including cognitive decline (36%), seizures (35%), headaches (30%), and neurological deficits like aphasia (20%) and motor deficits (20%) are relative common in patients with brain tumors (3, 4). Importantly, these symptoms often occur in clusters and may persist as long-term comorbidities even after surgical intervention (5–7), contributing to higher recurrence rates and poorer prognosis. Among these comorbidities, sleep disturbances are of growing concern due to their significant impact on caner progression and management. Studies reported higher prevalence of sleep disturbances in individuals with cancer (30% - 93.1%) compared to the general population (9% - 20.9%) (8–11), with brain tumor patients being particularly vulnerable (12, 13). Previous surveys indicated that 61.5% of brain tumor patients reported poor sleep quality 21.5% and insomnia symptoms (14), and 5% with excessive daytime sleepiness (EDS) (15). Structural factors such as tumor-induced compression or postoperative changes may damage key sleep–wake regulatory nuclei in the brain (16–18), leading to sleep disruption and a broad range of symptoms (19, 20). For instance, craniopharyngiomas in the saddle area can compress or infiltrate the hypothalamus, impairing hypothalamic secretions and inducing secondary narcolepsy. Given the critical role of sleep in physical and mental health, sleep disturbances can cause daytime dysfunction (19, 21, 22), accelerate tumor progression (13), reduce treatment efficacy, and negatively affect long-term prognosis (23).

Despite increasing recognition of sleep disturbances in patients with brain tumors, existing research remains limited to a few single-center, small-sample observational studies. Current knowledge of the relationship between brain tumors and sleep is fragmented, and the associated clinical characteristics and risk factors are not yet well-defined. To enhance the understanding and raise awareness of these co-occurring disturbances, we conducted a narrative review of recent evidence, offering a comprehensive synthesis of clinical findings and their connections.

## 2 Clinical characteristics of sleep disturbances in brain tumor patients

Sleep surveys have found that 57% to 81.8% of patients with brain tumor experience poor sleep quality (13, 15, 24–27) primarily due to various forms of sleep disturbances.

### 2.1 Insomnia

Current research identifies insomnia as the most prevalent sleep disturbance among patients with brain tumors, typically characterized by difficulties in initiating and maintaining sleep (28). In a Korean national cohort of 4,851 patients with malignant brain tumors (29), the preoperative prevalence of insomnia was 18.8%, with an additional 9.2% developing new-onset insomnia after surgery (29). A longitudinal study further indicated that insomnia was associated with increased post-operative mortality within two years, despite the duction of symptom severity following surgery (29). Persistent insomnia is reported in approximately 50% of patients with low-grade gliomas, pituitary adenomas, and recurrent gliomas (30–32). Its severity may worsen when accompanied by postoperative complications such as headaches and epilepsy (31) or as a side effect of treatments including corticosteroids and radiotherapy (33, 34). However, the specific pathophysiological links between insomnia and distinct brain tumor types remain insufficiently understood. Using the Athens Insomnia Scale, one study found a higher prevalence of insomnia in patients with malignant brain tumors (61.8%, n = 35) compared to those with benign brain tumors (54.3%, n = 68) (15). Tumor location, such as suprasellar versus non-suprasellar regions, did not significantly influence insomnia prevalence (64.5%, n = 31 vs. 61.1%, n = 67, respectively) (15). Cross-sectional studies also reported high rates of insomnia (46.8% - 59.2%) in untreated pituitary adenoma and meningioma patients (15, 27). In a prospective study of 70 patients awaiting tumor resection, those with bilateral tumors exhibited more severe insomnia (35). Objective assessments using polysomnography (PSG) remain rare. Small sample PSG studies in glioma patients have yielded inconsistent results regarding sleep onset latency (SOL) (36–38), a key marker of insomnia severity. Importantly, patients with malignant brain tumors are particularly vulnerable to pre- and post-operative anxiety and depression (15, 39, 40), factors strongly correlated with insomnia (24, 26). For example, in a cohort of 358 patients with glioma, while sleep quality briefly improved post-surgery, long-term anxiety about disease progression was linked to sleep disruption (41). Similarly, a pediatric study in post-operative medulloblastoma patients (n = 37), found that emotional distress was associated with longer SOL, more nocturnal awakenings, and reduced sleep efficiency (42). In contrast, such associations were not consistently observed in patients with benign brain tumors (28) warranting caution when generalizing findings across tumor types. Collectively, evidence suggests that insomnia is highly prevalent in brain tumor populations and may serve as a prognostic indicator, particularly in malignant cases. Its impact appears closely tied to severity and the presence of psychological comorbidities (28, 30, 43).

### 2.2 Excessive daytime sleepiness

EDS, also known as hypersomnolence disorder, is a significant sleep disturbance in patients with brain tumors, characterized by reduced alertness and episodes of unintended daytime sleep. It can critically impair cognitive functions, particularly attention and memory (44). Based on the Epworth Sleepiness Scale (ESS), one study reported that 4.9% (n = 103) of brain tumor patients experienced EDS, defined as an ESS score > 10 (15). EDS is most prevalent in patients with tumors affecting the basal ganglia, hypothalamus and brainstem, such as craniopharyngiomas and pituitary adenomas (34, 45). PSG and multiple sleep latency test (MSLT) studies have reported EDS prevalence rates ranging from 50% to 83% in this population (45–47). In pediatric cohorts, the incidence of secondary narcolepsy following childhood brain tumors is approximately 1.67% (n = 2,336) (34) with EDS as a primary symptom. Secondary narcolepsy is more common in tumors of the sellar region (48), with incidence rates between 14.3% and 35% among craniopharyngioma patients (46, 49). These tumors typically lead to reduced cerebrospinal fluid orexin concentrations, though levels often normalize following tumor resection (50–52). EDS may be exacerbated by hypothalamic involvement and associated weight gain, which can increase the risk of sleep apnea. Sleep apnea disrupts sleep macrostructure via hypoxemia, thereby intensifying EDS (49, 53). A notable case report documented marked improvement in EDS after surgical resection of a Grade II temporal lobe hippocampal glioma causing secondary narcolepsy, with ESS scores decreasing from 16 to 3 (54). In pediatric craniopharyngioma patients, postoperative hypothalamic damage may also impair nighttime melatonin secretion further contributing to EDS (53, 55). Current research on EDS in brain tumor patients largely focuses on the craniopharyngiomas and pituitary adenomas, underscoring the need for broader investigations across diverse tumor types (31).

### 2.3 Sleep-related breathing disorders

A meta-analysis of post-operative PSG in survivors of pediatric brain tumors revealed that approximately 64% had comorbid sleep-related breathing disorders (SBDs) (56). SBD is frequently associated with EDS in clinical settings, a pattern consistently observed in brain tumor patients. In one study of 31 pediatric brain tumor patients who developed postoperative EDS 58.1% met diagnostic criteria for SBDs (45). Notably, younger patients (0–12 years) were more affected, with 83.3% exhibiting an apnea-hypopnea index (AHI) > 1 event/hour compared to 40% of older patients (aged 12–18 years) with AHI > 5 events/hour. Among these 31 children, central sleep apnea was identified in 22.2% of cases, highlighting the importance of comprehensive SBD screening, particularly when tumors involve regions regulating respiratory control, such as the sellar area, hypothalamus, basal ganglia, and brainstem (57). In adult brain tumor patients, findings from the STOP-BANG questionnaire indicate that the severity of obstructive sleep apnea (OSA) is significantly correlated with poorer prognosis and higher readmission rates within 30- and 90-days post-surgery (58, 59).

### 2.4 Circadian arrhythmia and parasomnias

**Figure 1:**
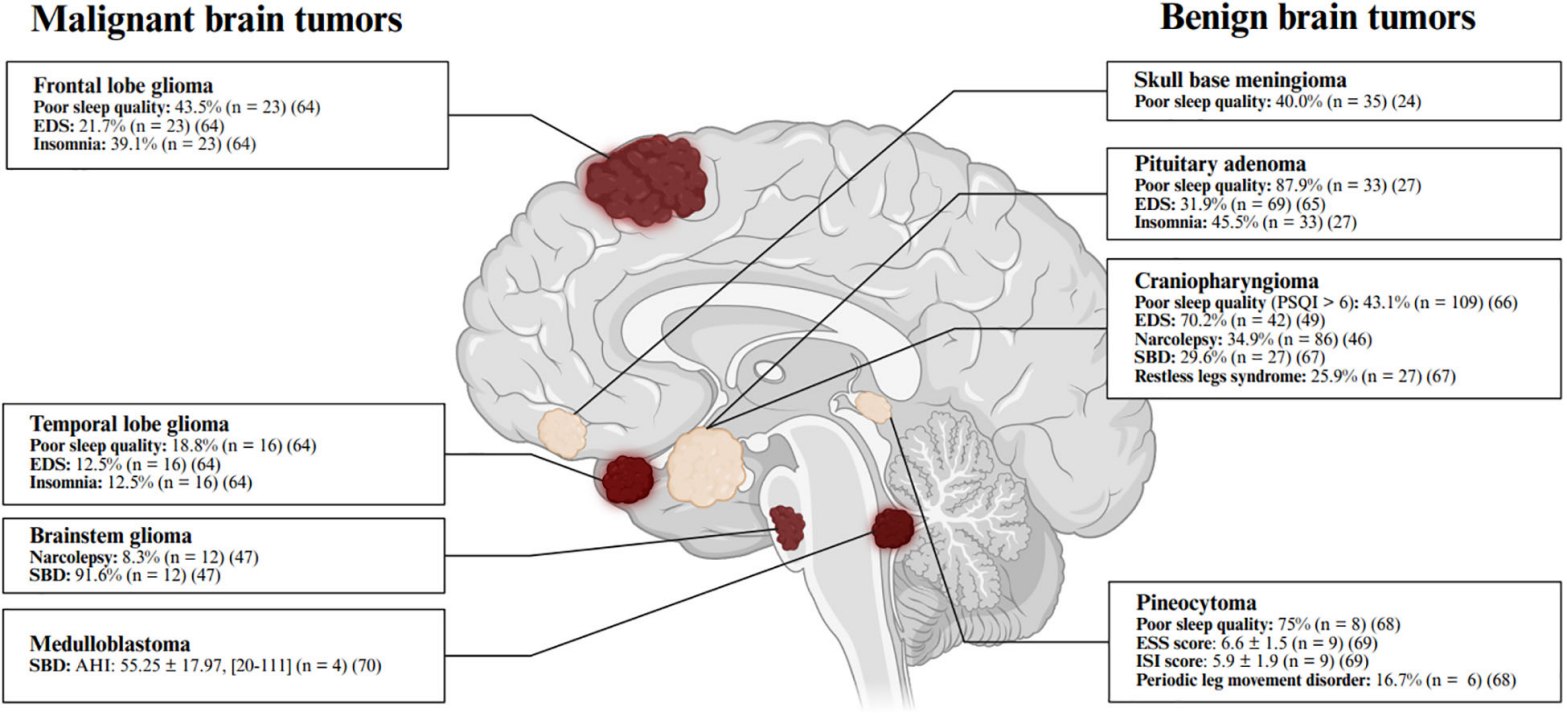
Commonly reported sleep disturbances in patients with brain tumors. The prevalence of sleep disturbances varies by tumor type (24, 27, 47, 49, 64–70), with specific rates and sample sizes detailed in the corresponding sections and summarized in this figure (64–70). Continuous variables are presented as mean ± standard error of the mean (SEM) and [range]. Poor sleep quality is defined as a Pittsburgh Sleep Quality Index (PSQI) score > 5. Excessive daytime sleepiness (EDS) is defined as an Epworth Sleepiness Scale (ESS) score > 10. Insomnia is indicated by an Insomnia Severity Index (ISI) score > 7. Narcolepsy is diagnosed based on polysomnography (PSG) and multiple sleep latency test (MSLT) results. Sleep-related breathing disorder (SBD) is diagnosed either by self-report or by an apnea-hypopnea index (AHI) > 5 events/hour. Periodic limb movement disorder (PLMD) is considered clinically significant when periodic limb movements exceed 10 per hour. Figure created with BioRender.com.

Circadian rhythm disturbances and rapid eye movement (REM) sleep behavior disorder (RBD) have also been reported in brain tumor patients and should be carefully distinguished from EDS. Actigraphy monitoring revealed circadian rhythm disruption in 70% (n = 35) of patients with malignant brain tumors and 57.7% (n = 68) benign brain tumors (19). Specifically 59.3% of untreated patients with pituitary tumors and meningiomas(n = 77), exhibited disrupted circadian patterns (25). These alterations are likely associated with lesions in the sellar region, which can impair melatonin secretion (19, 60). RBD, a parasomnia occurring during REM sleep, is characterized by dream-enacting behaviors and REM sleep without atonia on polysomnographic recordings (61). To date, brain tumor-associated has been reported primarily in case studies. For instance, one patient with brainstem lymphoma exhibited vivid dreams and violent behavior consistent with RBD (62), which markedly improved following surgical resection (62). A retrospective analysis of eight patients with brain tumors and RBD suggested that the disorder may be linked to tumor-related damage in the brainstem and limbic system (63). Since RBD is often a prodromal feature of α-synucleinopathy-related neurodegenerative diseases, the long-term risk of neurodegenerative risk in brain tumor patients with RBD remains unknown (**Figure 1**).

## 3 Risk factors and mechanism underlying sleep disturbances in brain tumor patients

### 3.1 Pre-existing sleep disorders

While brain tumors can lead to newly onset sleep disturbances - often linked to tumor location, type, or treatment modalities (13, 30–32, 42, 65–69) — patients may also present with pre-existing sleep disorders such as insomnia prior to diagnosis. One study reported that up to 62% of patients experienced sleep disturbances before their tumor was identified (13). As the tumor progresses, these pre-existing conditions may worsen, typically presenting as reduced total sleep time and increased nocturnal awakenings (71, 72).

### 3.2 Brain tumors and the intervention

Previous studies have shown that brain tumors can induce new-onset sleep disturbances, which may be associated with tumor characteristics (73) or therapeutic interventions (13, 30–32, 41, 74–77) such as surgery, radiotherapy, and medication. Brain tumor-related treatments, including surgical resection (30, 31, 41, 74), radiotherapy (75–77), and specific medications such as corticosteroid use (13, 32), can collectively disrupt sleep-wake regulation. Damage to the sleep-regulatory nuclei or neural pathways, or the neurotransmitters or hormone systems involved in sleep may result from both the tumor and its treatment, thereby increasing the risk of sleep disturbances (45). Neuroendocrine dysfunction is a key contributor to sleep disruption in brain tumor patients. For example, impairment of the suprachiasmatic nucleus can reduce melatonin secretion, altering circadian rhythms and degrading sleep quality (23). Hypothalamic involvement may lead to decreased orexin levels or dysregulation of the hypothalamic-pituitary-adrenal (HPA) axis, resulting in EDS and fragmented nighttime sleep (23, 53). In a study of postoperative craniopharyngioma patients (n = 15), elevated evening and nighttime salivary cortisol levels were associated with increased nocturnal awakenings and reduced total sleep time (23). Pituitary tumors can also disrupt anterior pituitary hormone secretion, potentially altering sleep architecture (78). Although no direct cases have been reported, elevated prolactin or thyroid-stimulating hormone (TSH) levels may contribute to sleep disruption (79, 80), while excess growth hormone can lead to soft tissue proliferation in the upper airway, increasing the risk of OSA (81, 82). Postoperative increases in SBDs have been observed in pediatric brain tumor patients, rising from 4.6% to 64% (15, 56), possibly reflecting impaired respiratory control following surgery (47). Radiotherapy further compounds sleep issues. In glioma patients (n = 68), over 90% reported EDS after radiotherapy, along with fatigue and reduced daytime function (33). These symptoms peaked at two weeks into treatment and persisted for up to 10 weeks, potentially linked to high radiation doses ( > 30 Gy) (34) or radiation-induced HPA axis impairment (83). Radiotherapy may also lead to upper airway muscle fibrosis and neuromuscular injury, increasing OSA risk (84). Insomnia also tends to increase immediately after radiotherapy and decline thereafter. In a study of patients with low-grade glioma and meningioma (n = 23), insomnia prevalence rose to 23.3% during radiotherapy and declined to 8.7% one month after completing proton therapy (85). Similar trends were observed in glioblastoma patients (86). Potential mechanisms include hypothalamic and pineal gland dysfunction, as well as radiotherapy-induced mood changes, pain, and fatigue, which can contribute to insomnia (87, 88). Corticosteroids, commonly used perioperatively to manage vasogenic edema, nausea, and pain, are potent modulators of the HPA axis (89). They stimulate wake-promoting neural nuclei and may activate the ascending reticular activating system, which can reduce sleep drive and disrupt sleep-wake balance. A study of recurrent glioma patients (n = 340) found that corticosteroid users had a significantly higher prevalence (58.5% vs. 38.4%) and severity of insomnia compared to non-users (32).

### 3.3 Emotional problems

Studies have demonstrated that anxiety and depression are more prevalent in brain tumor patients than in the general population (24, 26, 78). Assessments using the Hospital Anxiety and Depression Scale indicate that depressive symptoms are more severe in patients with malignant brain tumors compared to those with benign tumors (15, 39). Notably, these emotional disturbances are closely associated with changes in sleep patterns both before and after surgical intervention (24, 26). Research has shown that the severity of pre-treatment anxiety and depression positively correlates with the intensity of sleep disturbances in patients with primary brain tumors (15). In a cohort of 358 glioma patients, sleep quality showed temporary improvement shortly after surgery; however, anxiety about tumor recurrence subsequently worsened sleep quality and contributed to long-term insomnia-like symptoms (41). This phenomenon appears to be less pronounced in patients with benign brain tumors (15). In pediatric populations, similar findings have been reported. For example, studies in children with medulloblastoma found that depressive symptoms were associated with prolonged sleep latency, increased nocturnal awakenings, and reduced sleep efficiency (42).

### 3.4 Other comorbidities

Comorbid conditions such as headache and epilepsy are known to exacerbate cognitive deficits, reduce quality of life, and contribute to various sleep disturbances in brain tumor patients. Headache has been identified as an independent predictor of insomnia in this population (n = 100) (24). Additionally, seizures have been associated with EDS in pediatric brain tumor patients (n = 70) (90). Fatigue is another prevalent symptom in brain tumor patients and is strongly linked to sleep disturbances (91). In patients with glioma, reduced physical activity due to fatigue may further exacerbate sleep problems (92). As a result, fatigue and sleep disturbances frequently co-occur, particularly in patients with recurrent gliomas. In one study (n = 340), over 80% of patients with comorbid insomnia also reported symptoms of fatigue (32). Although the bidirectional mechanisms linking fatigue and sleep disturbances remain poorly understood, several studies suggest that HPA axis dysregulation may play a central role in both conditions (32, 37, 93).

## 4 Assessments and interventions for sleep disturbances in brain tumor patients

### 4.1 Assessments

Sleep disturbances in individuals with brain tumors are typically assessed using both questionnaires and PSG, offering a comprehensive evaluation from subjective and objective perspectives. PSG is widely recognized as the gold standard for sleep assessment, enabling detailed evaluation of nocturnal sleep and daytime sleepiness (44, 94). As such, PSG is considered the primary diagnostic tool for identifying sleep disorders in brain tumor patients, particularly SBD, secondary narcolepsy, and RBD. Although no specific sleep questionnaire developed for brain tumor patients, the Pittsburgh Sleep Quality Index (PSQI) (n = 205, Cronbach’s alpha = 0.79) (95), Insomnia Severity Index (ISI) (n = 1,026, Cronbach’s alpha = 0.92) have demonstrated strong reliability and validity in cancer populations (96) and widely used in the sleep research in patient with cancers (15, 26). As well, the ESS showed great sensitivity and specificity in craniopharyngioma patients (97). Additionally, actigraphy is a non-invasive method for monitoring res and activity cycles, which could provide objective data on sleep duration and patterns. This is especially valuable for detecting circadian rhythm sleep–wake disorders in brain tumor patients.

### 4.2 Interventions

Multiple clinical interventions, both non-pharmacological and pharmacological, have shown effectiveness in managing sleep disturbances among brain tumor patients. The primary treatment strategy should involve early identification and, when possible, removal of the underlying cause, followed by targeted interventions.

### 4.3 Non-pharmacological approaches

Particularly, sleep hygiene education and cognitive behavioral therapy are recommended as first-line treatments in cancer populations to improve sleep-related cognition and behavior (98). Cognitive behavioral therapy for insomnia (CBT-I) has been validated in brain tumor patients, demonstrating efficacy in improving sleep and offering additional benefits such as reduced fatigue, depression, and anxiety, thereby enhancing quality of life (87, 88, 99). However, the long-term efficacy of CBT-I remains uncertain (100, 101). For patients with EDS, modified CBT-I protocols that emphasize sufficient nighttime sleep and structured daytime napping may be beneficial (48, 102). Given the critical role of sleep in cancer prognosis and recovery (103), CBT-I should be implemented thoughtfully and personalized to meet individual patient needs (104).

### 4.4 Pharmacological management of insomnia

Pharmacological treatments for insomnia in the general population include non-benzodiazepines, benzodiazepines, and melatonin receptor agonists (98, 105). Among cancer patients, non-benzodiazepine hypnotics, such as zopiclone, eszopiclone, and zolpidem, are often preferred due to their relatively mild side effect profiles (106). However, no randomized controlled trials (RCTs) have specifically investigated their efficacy in brain tumor patients. Still, studies in broader oncology populations suggest that sequential treatment involving cognitive behavioral therapy followed by zolpidem may be effective for long-term insomnia remission (107, 108). Zopiclone (3.75–7.5 mg) and eszopiclone (3 mg) have been shown to reduce SOL, increase total sleep time, and improve subjective sleep satisfaction in patients with other cancers (108–110). A small study reported that zolpidem (10 mg) improved insomnia symptoms in brain tumor patients (n = 7) (111); however, caution is warranted, as chronic use exceeding 300 mg/year has been associated with increased risks of oral cancer and mortality (112–114). Benzodiazepines are generally not recommended for brain tumor patients due to their potential to worsen OSA, EDS, and cognitive dysfunction (115–118). Melatonin supplementation has demonstrated efficacy in managing insomnia related to pineal tumors (119), while low-dose antidepressants such as mirtazapine and trazodone have proven beneficial in cancer patients more broadly, though data specific to brain tumors are lacking (120, 121). The potential role of selective orexin receptor antagonists remains unexplored in this population and presents a promising area for future research (122).

### 4.5 Pharmacological management of EDS

Managing EDS in brain tumor patients requires an etiology-focused approach, beginning with surgical resection to eliminate the underlying cause. Pharmacological options include stimulant medications such as modafinil, pitolisant, and methylphenidate. Pitolisant has shown efficacy in treating EDS, cataplexy, and hypnagogic hallucinations, particularly in craniopharyngioma patients with secondary narcolepsy (20, 49, 123). Modafinil has demonstrated modest benefits, particularly in cognitive functioning, although its overall utility in brain tumor patients remains limited (118, 124). Postoperative melatonin administration in craniopharyngioma patients has also shown promise in reducing EDS (55).

### 4.6 Management of SBD

SBD is frequently observed in patients with brainstem tumors or craniopharyngiomas, where surgical resection often leads to substantial symptom improvement (125). For persistent SBD after surgery, continuous positive airway pressure (CPAP) therapy is the first-line management (20, 36, 125, 126). Polysomnographic evaluations should be conducted both pre- and postoperatively to accurately diagnose and monitor SBD.

### 4.7 Exercise-based interventions

Moderate to high-intensity aerobic and resistance training may improve sleep quality, physical fitness, and mental well-being in brain tumor patients (127). However, findings are mixed. For example, one study of high-grade glioma patients undergoing postoperative radiotherapy found that regular aerobic exercise (yoga, 2–3 sessions/week, 60 minutes/session) significantly improved sleep quality (128). Conversely, a Danish RCT found no improvement in insomnia, EDS, or quality of life following a 6-week intensive physical therapy program (3 sessions/week, 90 minutes/session) (129). Longer-duration programs may offer greater benefit. A 6-month home-based aerobic exercise intervention (20–45 minutes/session, 3 times/week) monitored by physical therapists improved sleep quality (reduced PSQI scores), cardiopulmonary function, and cognitive performance in glioma patients (130). Despite inconsistent outcomes, structured exercise under professional guidance is generally recommended for this population.

## 5 Summary and future perspective

In the context of brain tumors, the symptomatology of sleep disturbances is complex, yet their underlying etiologies remain poorly understood. To date, a tumor feature–oriented sleep phenotype framework has not been established. However, current evidence indicates that insomnia is the most commonly reported sleep disorder in brain tumor patients, while EDS, particularly secondary narcolepsy, is frequently associated with tumors located in the sellar and thalamic regions. SBD also occurs in this population, and the use of PSG and CPAP therapy is strongly recommended during both pre- and postoperative care.

Given the substantial impact of poor sleep on quality of life and long-term prognosis, early identification and appropriate intervention are essential. Due to the limited accessibility and financial burden of PSG, consumer-grade portable devices (e.g., wearables, home electroencephalogram systems) offer promising alternatives for sleep monitoring, owing to their cost-effectiveness and ease of use (131, 132). Furthermore, validated digital questionnaires integrated with machine learning algorithms may provide efficient tools for initial sleep disturbance screening in routine clinical care (133). However, notable accuracy gaps persist between consumer devices and PSG in sleep staging (134).

Artificial intelligence (AI) represents a transformative area in sleep medicine. Emerging algorithms can analyze complex multimodal data from wearable sensors and electronic health records to improve the prediction and classification of sleep disorders. Previous studies have shown that AI models can accurately detect OSA using oximetry and photoplethysmography signals (135), or respiratory vibration signals from wearables (136). Nonetheless, the lack of standardized validation frameworks across populations and devices, along with ethical, safety, and legal concerns, remains a major barrier to clinical adoption (137, 138). Moreover, our current understanding of the mechanisms underlying sleep-wake dysregulation in brain tumor patients is fragmented, limiting the development of targeted treatments.

## 6 Limitations

This review has several limitations. Unlike previous systematic reviews on sleep in brain tumor patients (139–141), we did not formally assess the quality of evidence, as this study followed a narrative review format. Additionally, we did not stratify findings by age groups or tumor types due to the scarcity and heterogeneity of available data. Instead, we adopted an approach focused on specific sleep disorder types and their associated risk factors.
